# ^1^H Magnetic Resonance Spectroscopy of live human sperm

**DOI:** 10.1093/molehr/gax025

**Published:** 2017-05-23

**Authors:** S Reynolds, S J Calvert, M N Paley, A A Pacey

**Affiliations:** 1 Academic Unit of Radiology, Department of Immunity, Infection and Cardiovascular Disease, University of Sheffield, Sheffield, S10 2JF, UK; 2 Academic Unit of Reproductive & Developmental Medicine, Department of Oncology and Metabolism, University of Sheffield, Level 4, The Jessop Wing, Tree Root Walk, Sheffield, S10 2SF, UK

**Keywords:** metabolomics, human sperm, sperm washing, Magnetic Resonance Spectroscopy

## Abstract

**STUDY QUESTION:**

Can ^1^H Magnetic Resonance Spectroscopy (MRS) be used to obtain information about the molecules and metabolites in live human spermatozoa?

**SUMMARY ANSWER:**

Percoll-based density gradient centrifugation (DGC) followed by a further two washing steps, yielded enough sperm with minimal contamination (<0.01%) from seminal fluid to permit effective MRS which detected significant differences (*P* < 0.05) in the choline/glycerophosphocholine (GPC), lipid and lactate regions of the ^1^H MRS spectrum between sperm in the pellet and those from the 40%/80% interface.

**WHAT IS KNOWN ALREADY:**

Current methods to examine sperm are either limited in their value (e.g. semen analysis) or are destructive (e.g. immunohistochemistry, sperm DNA testing). A few studies have previously used MRS to examine sperm, but these have either looked at seminal plasma from men with different ejaculate qualities or at the molecules present in pooled samples of lyophilized sperm.

**STUDY DESIGN, SAMPLES/MATERIALS, METHODS:**

Sperm suspended in phosphate buffered saline (PBS) at 37°C were examined by ^1^H MRS scanning using a ^1^H excitation-sculpting solvent suppression sequence after recovery from fresh ejaculates by one of three different methods: (i) simple centrifugation; (ii) DGC with one wash; or (iii) DGC with two washes. In the case of DGC, sperm were collected both from the pellet (‘80%’ sperm) and the 40/80 interface (‘40%’ sperm). Spectrum processing was carried out using custom Matlab scripts to determine; the degree of seminal plasma/Percoll contamination, the minimum sperm concentration for ^1^H MRS detection and differences between the ^1^H MRS spectra of ‘40%’ and ‘80%’ sperm.

**MAIN RESULTS AND THE ROLE OF CHANCE:**

DGC with two washes minimized the ^1^H MRS peak intensity for both seminal plasma and Percoll/PBS solution contamination while retaining sperm specific peaks. For the MRS scanner used in this study, the minimum sperm concentration required to produce a choline/GPC ^1^H MRS peak greater than 3:1 signal to noise ratio (SNR) was estimated at ~3 × 10^6^/ml. The choline/GPC and lactate/lipid regions of the ^1^H spectrum were significantly different by two-way ANOVA analysis (*P* < 0.0001; *n* = 20). ROC curve analysis of these region showed significant ability to distinguish between the two sperm populations: choline/GPC ROC AUC = 0.65–0.67, lactate/lipid ROC AUC = 0.86–0.87.

**LIMITATIONS, REASONS FOR CAUTION:**

Only 3–4 semen samples were used to assess the efficacy of each sperm washing protocol that were examined. The estimated minimum sperm concentration required for MRS is specific to the hardware used in our study and may be different in other spectrometers. Spectrum binning is a low resolution analysis method that sums MRS peaks within a chemical shift range. This can obscure the identity of which metabolite(s) are responsible for differences between sperm populations. Further work is required to determine the relative contribution of somatic cells to the MRS spectrum from the ‘40%’ and ‘80%’ sperm.

**WIDER IMPLICATIONS OF THE FINDINGS:**

^1^H MRS can provide information about the molecules present in live human sperm and may therefore permit the study of the underlying functional biology or metabolomics of live sperm. Given the relatively low concentration of sperm required to obtain a suitable MRS signal (~3 × 10^6^/ml), this could be carried out on sperm from men with oligo-, astheno- or teratozoospermia. This may lead to the development of new diagnostic tests or ultimately novel treatments for male factor infertility.

**STUDY FUNDING AND COMPETING INTEREST(S):**

This work was supported by the Medical Research Council Grant MR/M010473/1. The authors declare no conflicts of interest.

## Introduction

Poor sperm quality is a major barrier to conception and is thought to contribute to 30–50% of cases of infertility in heterosexual couples (Pacey, 2009). However, our knowledge of sperm dysfunction is limited and the value of current laboratory tests has been questioned (Tomlinson, 2016). The techniques of semen analysis rely upon visual identification of spermatozoa by microscopy to estimate sperm concentration and the proportion of sperm with progressive motility and ideal morphology (WHO, 2010). However, although these techniques have been subject to regular review, they remain largely similar to those developed by Macleod (1956) and have advanced very little since that time. To date, few adequate tests have been developed and brought into routine clinical practice (De Jonge, 2012) and those that have (e.g. sperm DNA damage testing) are typically destructive to sperm. Therefore, there is a need for semen analysis to be complemented with more specific sperm function tests that examine various aspects of sperm biology and provide information about the etiology that cause them to swim badly or have poor size and shape.

Metabolomic studies by Magnetic Resonance Spectroscopy (MRS) have been used in many biological systems to provide insight into functional aspects of cell cultures, tissues and bio-fluids (Emwas *et al.*, 2013). For example, MRS has been used to examine the molecular composition of seminal plasma (Lynch *et al.*, 1994; Hamamah *et al.*, 1998; Sharma *et al.*, 2001; Gupta *et al.*, 2011a,b; Jayaraman *et al.*, 2014) and concluded that it might be possible to discriminate some phenotypes of poor semen quality to a high degree of accuracy. However, other studies in the turbot (Dreanno *et al.*, 2000), goat (Patel *et al.*, 1998, 1999), boar (Marin *et al.*, 2003), rhesus macaque (Hung *et al.*, 2009; Lin *et al.*, 2009) and human (Paiva *et al.*, 2015) have each used MRS to examine sperm. But in each case the sperm were prepared for MRS by a variety of methods to extract metabolites or other cellular material for scanning, thereby killing them. For example, in the study by Paiva *et al.* (2015) washed sperm from ejaculates with a variety of phenotypes were subjected to a methanol extraction before being lyophilized for MRS. While other analytical techniques have been applied to the study of sperm (e.g. Mass Spectrometry (Paiva *et al.*, 2015) and Raman spectroscopy (Huser *et al.*, 2009)), to our knowledge no one has so far reported the use of MRS on samples of live human sperm. Therefore, we hypothesized whether MRS could provide a non-destructive method to obtain additional functional and quantitative information about live sperm metabolites and thereby provide a potential avenue for the development of new diagnostic tests or novel treatments for male factor infertility.

While there are a variety of different approaches to conduct MRS, the ^1^H nucleus is preferred as it is ubiquitously abundant and provides the best signal to noise per unit time. However, a multitude of resonant peaks and a narrow chemical shift range can result in spectral overcrowding, where individual peaks stack on top of one another masking their true size and position. Moreover, any contamination from biological fluids or material used in the preparation of samples for scanning can exacerbate spectral ‘crowding’ of a sample, potentially distorting the true concentration and obscuring peaks of interest. Since sperm are ejaculated in seminal plasma, a major challenge is how to obtain a sufficiently pure population of live sperm to examine metabolic differences between them without contamination from seminal plasma or sperm preparation media used to isolate them. Consequently, the aim of this study is to address three questions: (a) What sperm preparation methods are suitable to prepare live sperm for ^1^H MRS analysis?; (b) What is the minimum concentration of sperm required for ^1^H MRS analysis using a commonly available spectrometer?; and (c) Are there differences in the ^1^H spectra from populations of sperm recovered from density gradient centrifuge (DGC) that could give important new biological information about their function?

## Materials and Methods

### Semen sample donation and analysis

For experiments to determine the best sperm washing method, semen samples were obtained from healthy volunteers following 2–3 days of sexual abstinence. All samples used in questions (a) and (b) were produced by masturbation at home and collected into a sterile plastic container (Sarstedt, Leicester, UK) before being delivered to the laboratory within 1 h. Upon arrival, an assessment of semen volume, sperm concentration and motility was performed on each sample according to World Health Organisation (WHO, 2010) methods. For some experiments, an additional assessment of sperm motility was completed with version 5.0 of the Sperm Class Analyzer (Microptic SL, Barcelona, Spain) using a Microtec LM-2 Microscope (Mazurek Optical Services Ltd, Southam, UK) and a Basler A312fc camera (Basler AG, Ahrensburg, Germany). In such cases, 3 μl of homogenous liquefied semen was placed in a Leja disposable counting chamber (Leja Products, Nieuw Vennep, The Netherlands) and a total of 500 sperm or five fields of view observed using a PLAN PH2 20x/0.40 infinite/0.17 objective lens to measure the proportion of progressive, non-progressive and immotile sperm according to the standard definitions of the software. All procedures for the recruitment of volunteers and the delivery of samples were approved by the University of Sheffield Research Ethics Committee (Ref: SMBRER293; Approved on 28.02.14) and all volunteers gave informed written consent for their samples to be used for the experiments described in this paper. Semen samples used to identify possible differences in ^1^H MRS spectra between the different populations of sperm recovered from DGC (question c) were obtained from men attending the Andrology Laboratory (Jessop Wing, Sheffield Teaching Hospitals NHS Foundation Trust, Sheffield, UK) for diagnostic semen analysis as part of infertility investigations. All samples were produced on-site and men gave their consent for any remaining semen to be used in this study once the routine semen analysis had been completed. Only those samples with sperm concentration and motility above WHO (2010) thresholds were used in these experiments and the assessment of sperm concentration and sperm motility were determined as described above. Ethical approval for this part of the study was given by the North of Scotland Research Ethics Service (Reference: 16/NS/009) with clinical governance provided by Sheffield Teaching Hospitals NHS Foundation Trust (Reference: STH19095).

### Sperm preparation techniques

**Figure 1:**
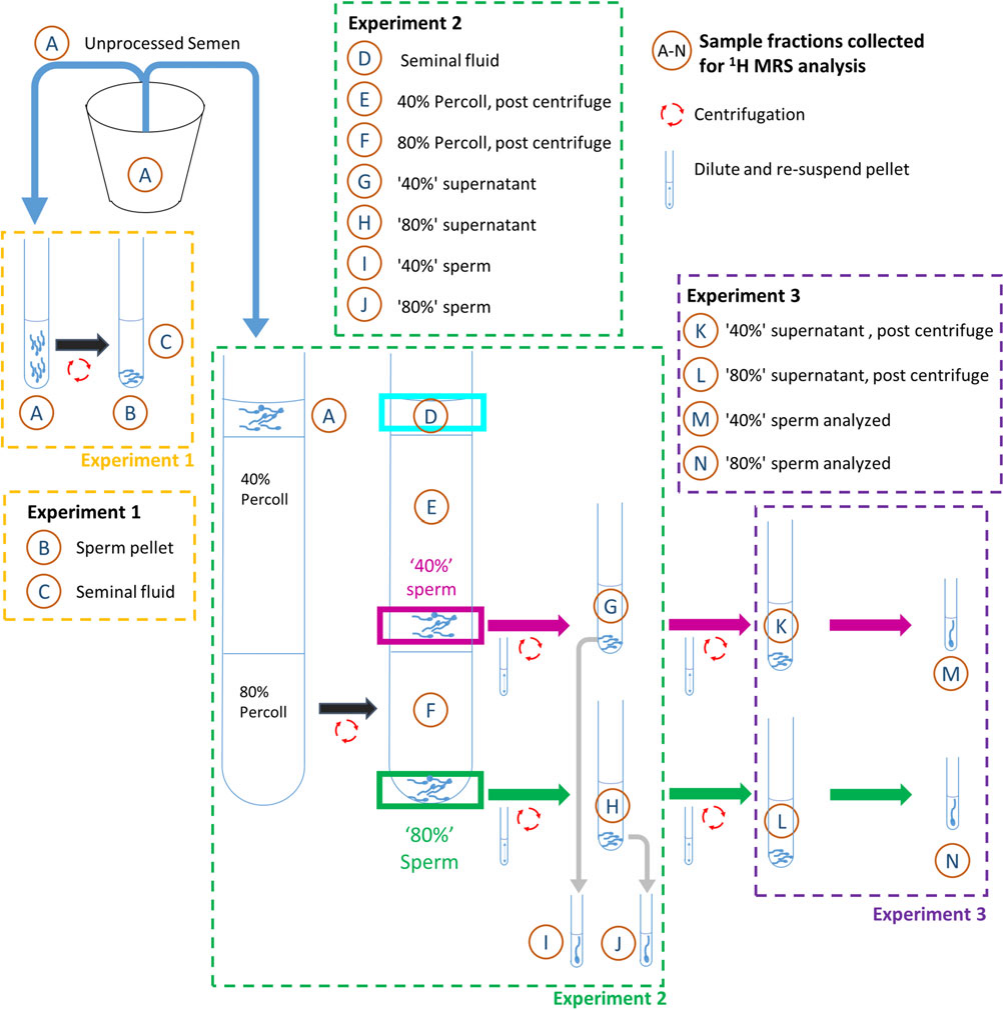
Overview of washing steps used for Experiments 1, 2 and 3 in the preparation of sperm samples for ^1^H MRS analysis. Fraction A is the donor semen sample. Fractions B and C obtained from Experiment 1; samples D–J obtained from Experiment 2; samples K–N obtained from Experiment 3 after performing the steps in Experiment 2. Drawings are not to scale, see Materials and Methods section for full details.

To obtain matched samples of (i) unprocessed semen; (ii) sperm-free seminal plasma; and (iii) seminal plasma free populations of sperm, for ^1^H MRS, three techniques of sperm preparation were used and described below. See Fig. 1 for identification of sample fractions A–N.

### Experiment 1: simple centrifugation

1 ml aliquots of each liquefied semen sample (Fraction A) were transferred to a 15 ml centrifuge tube (Biologix, Dutscher Scientific Ltd, Brentwood, UK) and centrifuged at 500 g for 15 min in a Sigma 3–16 K (SciQuip Ltd, Wem, UK). After centrifugation, the (upper) seminal plasma supernatant (Fraction C) was aspirated and transferred to a clean tube, whereas the sperm pellet (Fraction B) was re-suspended in approximately 600 μl phosphate buffered saline (PBS). Aliquots of each fraction were then taken for MRS analysis as described below.

### Experiment 2: DGC

Density gradients were prepared by carefully layering 1.8 ml of a 40% (v/v) isotonic solution of Percoll (GE Healthcare Life Sciences, Little Chalfont, UK) in phosphate buffer saline over 1.4 ml of an 80% (v/v) isotonic Percoll in PBS solution as described in (Elder and Dale, 2000). To estimate the amount of seminal plasma contamination in the washed sperm preparations 200 mM fumarate was added to each unprocessed semen sample (a 1:4 dilution according to semen volume) to give a final concentration of ~50 mM. Fumarate was chosen as its single resonance at 6.51 ppm did not overlap with other peaks in the semen spectrum. Since the addition of fumarate increased the volume of the unprocessed semen sample by about one-third, as well as changing its viscoelastic properties, sperm motility was reassessed (as outlined above) after fumarate addition. To each Percoll gradient, 1 ml of the semen-fumarate mixture (Fraction A) was carefully layered on top of the gradient before being centrifuged at 300 g for 20 mins. After centrifugation, the sperm recovered from the 40%/80% interface (termed ‘40%’ sperm for the purposes of this paper) and the sperm recovered from the pellet below the 80% Percoll (termed ‘80%’ sperm) were collected and transferred to separate tubes. PBS was added to each recovered fraction, 1 in 4 diluted, and the sperm re-suspended, followed by centrifugation at 500 g for 10 min. A sample from each of the fractions through the washing process (Fractions A and D–H) were retained for ^1^H MRS analysis in order to track the fumarate concentration at each stage. The recovered ‘40%’ sperm (Fraction I) and ‘80%’ sperm (Fraction J) were collected and re-suspended in 600 μl PBS and aliquots taken for ^1^H MRS analysis as described below.

### Experiment 3: DGC with an additional centrifugation step

To assess the extent of seminal plasma and/or Percoll contamination in the ‘40%’ and ‘80%’ sperm populations recovered from the standard DGC method (Experiment 2 outlined above), a series of further experiments were performed in which an additional wash step was included (Fig. 1). Briefly, ‘40%’ and ‘80%’ sperm populations were recovered from the initial 40%/80% (v/v) Percoll Gradient as for Experiment 2 before being 1 in 4 diluted with PBS and centrifuged again at 500 g for 10 min. The supernatant was aspirated and the pellets (Fractions I and J) were then re-suspended in 1.2 ml PBS after which 600 μl was taken for MRS analysis (400 μl used for MRS sample, with the remainder used for sperm concentration and motility measurements). The remaining 0.6 ml aliquot was re-centrifuged at 500 g for 20 min, to recover as many sperm as possible. After this step, the supernatants were aspirated and kept for ^1^H MRS analysis (Fractions K and L) and the final pellets were re-suspended with PBS added to yield a final volume of 600 μl (Fractions M and N) before also being analyzed by ^1^H MRS.

Following sperm preparation by each of the above methods, sperm concentration and sperm motility was re-measured in each sample according to WHO (2010) methods in order to calculate the efficiency of sperm preparation or use the data in MRS analysis as described below.

### MRS experiments

For each of the fractions recovered from the sperm washing steps illustrated in Fig. 1 a 350 μl aliquot was added to a 5 mm MRS tube (Norell, Morganton, NC, USA) along with 20 μl of D_2_O (Sigma Aldrich, Gillingham, UK). Sample tubes were kept at room temperature until they could be scanned. All samples were scanned, in a random order, at 37°C, with up to 10 min equilibration time, using a 9.4 T Bruker Avance III MRS spectrometer (Bruker BioSpin GmbH, Karlsruhe, Germany), with a 5 mm broadband observe probe. Each spectrum was acquired using a ^1^H water-gate, excitation sculpting, solvent suppression sequence (Spectral Width = 20 ppm, Number of acquisitions = 1024, Acquisition Time = 0.5 s, Repeat Time = 4 s, Time Domain Points = 8222). Each spectrum was acquired and processed using Bruker Topspin v2.1 software to produce a phase and baseline corrected spectrum. All spectra were referenced to the ^1^H lactate signal at a frequency offset δ = 1.33 ppm.

### Data analysis

Spectra were analyzed using custom Matlab scripts (Mathworks, Natick, MA, USA). A noise region with no visible peaks was chosen between 10–11 ppm and all spectra were normalized so that the intensity here had a median value of zero and an interquartile range (IQR) of 1. Spectra from composite fractions (e.g. seminal plasma, Percoll or sperm) were then fitted to a target spectrum to quantify the proportions of these in samples (e.g. the proportion of seminal plasma in washed sperm). A spectral binning method was then used to split the spectrum into 0.04 ppm regions which were integrated to yield a bin area. Estimates for amounts of a component within a spectrum (e.g. amount of residual seminal plasma (Fraction B) in washed sperm (Fraction C) in Experiment 1), were generated by dividing the sum of fitted spectrum bin integrals for the target spectrum by the same value for the spectrum being fitted. This is outlined in more detail in the Supplementary Material provided.

Metabolite bin integrals were compared against measured sperm concentrations in 31 matched samples of ‘40%’ and ‘80%’ sperm (Fractions I and J) to determine which metabolite peaks correlated with number of sperm. First, all spectra were normalized as above and binned at 0.04 ppm, generating 193 bins in total after removing the bins associated with the water peak between 4.5 and 5.2 ppm. A Pearson's correlation coefficient was calculated for each bin versus sperm concentration and spectral bins with *r*^2^ > 0.25 and a significance *P* < 0.01 were considered to be metabolite peaks from sperm. Bin locations containing a significant correlation were then used to determine the minimum sperm concentration required to yield an observable ^1^H MRS peak within each bin with a signal to noise ratio (SNR) > 3:1 (see Supplementary Material for details).

Differences in ^1^H MRS spectra from ‘40%’ and ‘80%’ sperm populations were examined using the protocol detailed in Experiment 3 (Fractions M and N, *n* = 20). Spectra were processed, normalized and binned at 0.04 ppm as described above for metabolite bin integrals versus sperm concentration. The binned spectral integrals were then normalized to sperm concentration so that differences between ‘40%’ and ‘80%’ sperm spectra could be compared using a two-way ANOVA with a Bonferroni multiple comparison test, where *P* < 0.05 was regarded as significant. Bin regions identified by the two-way ANOVA as significantly different were then subject to a receiver operator curve (ROC) calculation to examine the specificity and sensitivity of the bin to discriminate sperm from the ‘40%’ or ‘80%’ populations.

Data for progressive sperm motility and sperm concentration in matched samples were compared using Wilcoxon signed rank test using Matlab. Statistical differences between multiple groups were tested in Matlab using a Kruskal–Wallis non-parametric ANOVA test and a multi-comparison Bonferroni post-hoc test with (*P* ≤ 0.05) used as the significance threshold. All results are quoted as mean ± SEM unless otherwise stated.

## Results

### Selection of sperm washing method

#### Experiment 1: simple centrifugation

**Figure 2:**
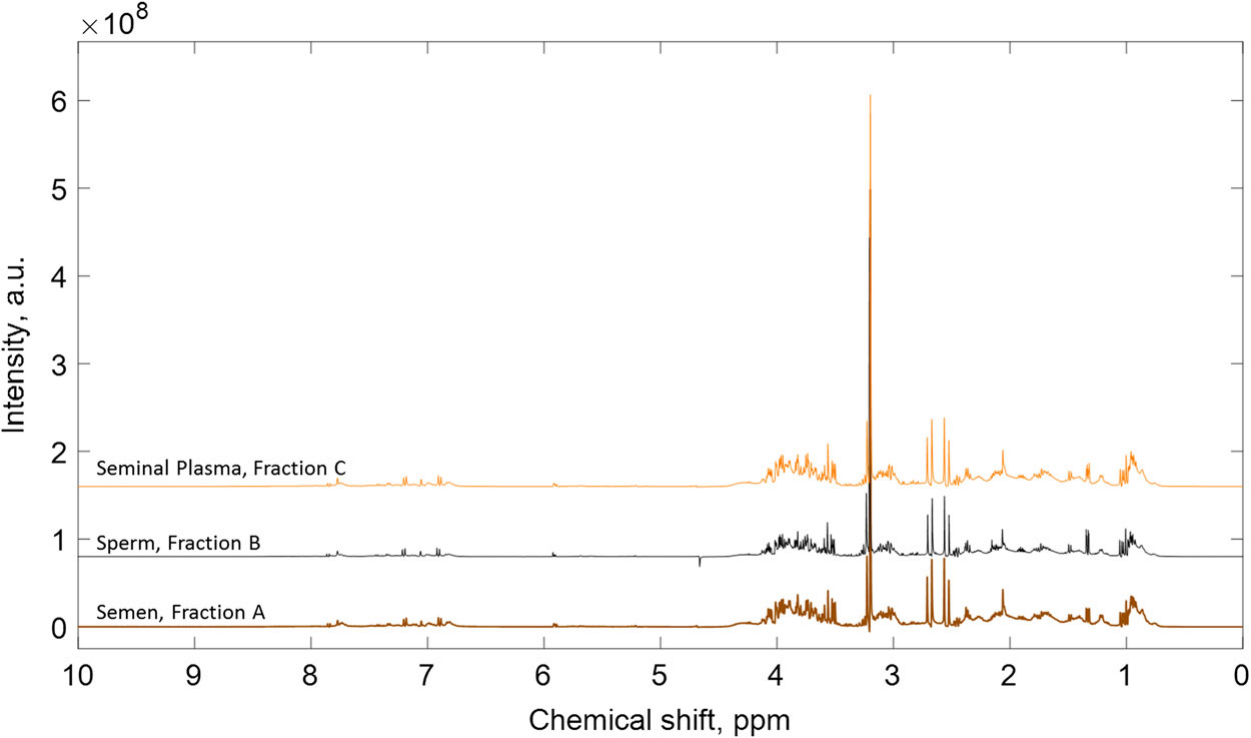
Representative ^1^H Magnetic Resonance Spectroscopy (MRS) spectra derived from donor semen after simple centrifugation (Experiment 1). Top, seminal plasma spectrum from Fraction C; middle, sperm spectrum from Fraction B; bottom, semen spectrum from Fraction A. See Fig. 1 for identification of each fraction component.

Figure 2 shows representative ^1^H MRS spectrum for (i) unprocessed semen (Fraction A); (ii) the sperm pellet (Fraction B); and (iii) seminal plasma (Fraction C); obtained from a single semen sample processed using the simple centrifugation method of sperm washing. Visually the spectra obtained for sperm and seminal plasma were very similar to the unprocessed semen and fitting the seminal plasma and sperm spectra to the unprocessed semen spectrum estimated the relative proportions of these components as 84 ± 9% and 10 ± 6%, respectively (sum of bin integrals, *n* = 4 see Supplementary Material for method details and example Supplementary Fig. S1). Conversely, a significant amount of seminal plasma (Fraction C) was still present in the spectrum from washed sperm (Fraction B): 91 ± 6% of the sperm spectrum (sum of bin integrals, *n* = 4; see example Supplementary Fig. S2). Although this method of sperm washing was successful at recovering sperm (retaining 46 ± 16% of the total sperm and 27 ± 5% of initial sperm progressive motility of the unprocessed-ejaculate) it was clearly inadequate to produce a seminal plasma free populations of sperm for ^1^H MRS scanning.

#### Experiment 2: DGC

**Figure 3:**
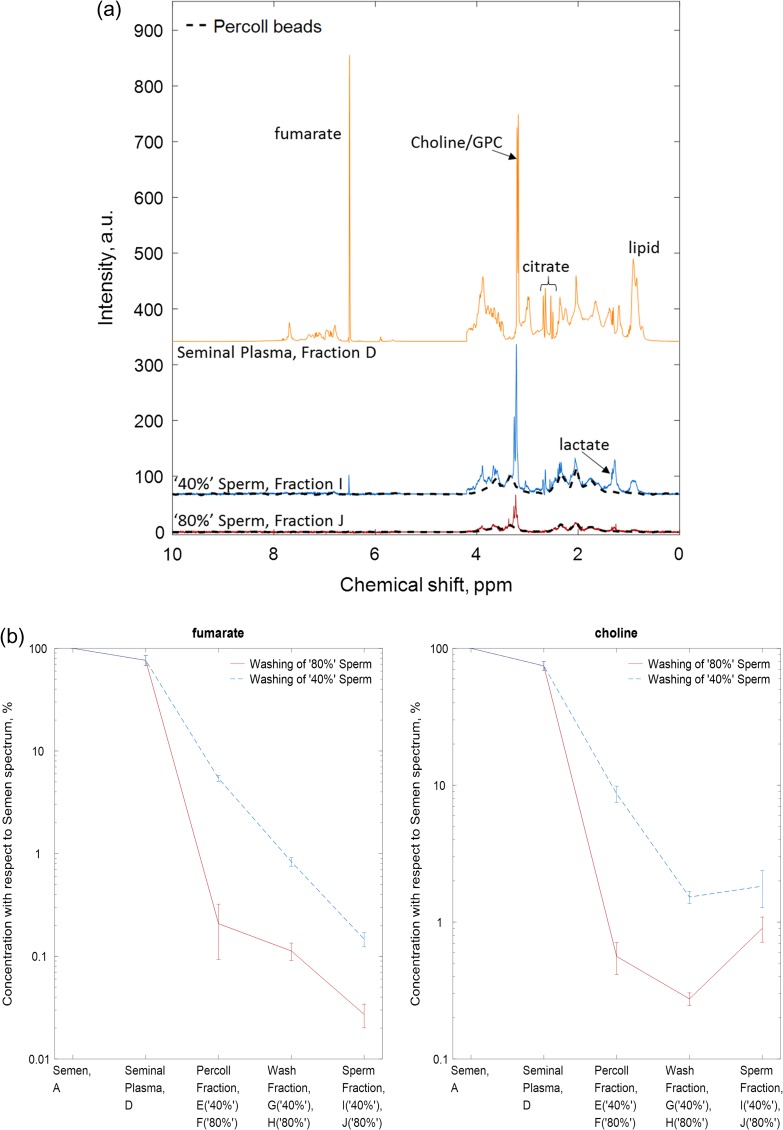
(**a**) Representative ^1^H MRS spectra derived from fumarate (200 mM) spiked donor semen after Percoll density centrifugation (Experiment 2); 80% sperm spectrum (Fraction J); 40% sperm spectrum (Fraction I); seminal plasma (Fraction D) spectrum recovered from supernatant after washing. ^1^H spectrum of Percoll beads colloidal solution (black dashed line) overlaid onto 80% and 40% sperm spectra. GPC, glycerophosphocholine. (**b**) The integrals for fumarate (left) and choline (right) from each of the fractions collected during the washing procedure, Fractions A, D–J. The fumarate and choline integrals were normalized with respect to their initial value from the semen spectrum. 80% sperm washings (red solid line), 40% sperm washings (dashed blue line) show the concentration the respective metabolite at each stage of the washing protocol. See Fig. 1 for fraction identification.

To obtain ^1^H MRS spectra from washed sperm with lower levels of seminal plasma contamination DGC was performed. A 40%/80% Percoll gradient diluted in PBS was used for the DGC and Fig. 3a shows the typical spectra obtained from ‘40%’ sperm (Fraction I), ‘80%’ sperm (Fraction J), seminal plasma (Fraction D) and that obtained from a Percoll beads colloidal solution diluted in PBS. A visual comparison of the spectra now clearly showed some distinct differences between ‘40%’ and ‘80%’ sperm as well as between both sperm populations and seminal plasma. The DGC method retained 20 ± 3% of the sperm (total of ‘80%’ and ‘40%’ sperm) and 55 ± 14% of the progressive motility (average for ‘40%’ and ‘80%’ sperm) compared to the initial values in unprocessed semen.

Although the DGC method using Percoll/PBS reduced the level of seminal plasma present in the spectra obtained from the two sperm populations, there was clearly more seminal plasma contamination in the ‘40%’ sperm compared to the ‘80%’ (Fig. 3a). Therefore, to estimate the amount of seminal plasma contamination, the fumarate peak was integrated in each sperm and sperm wash spectra, Fractions A and D to J, and normalized to its initial value in unprocessed semen. The concentration of fumarate in the seminal plasma spectrum, Fraction C, was 50 ± 1 mM. Based on this value the concentration of choline and citrate, two other major metabolites in semen, were estimated to be present at 37 ± 8 mM and 61 ± 21 mM, respectively. The mean fumarate integral in the spectra from both ‘80%’ and ‘40%’ sperm populations was diluted to 0.03 ± 0.01% and 0.15 ± 0.02% (*n* = 4), respectively, compared to its starting value (Fig. 3b). This is equivalent to a dilution of seminal plasma components of >600 for the ‘40%’ and >3000 for the ‘80%’ sperm populations. Similarly, the peak for choline at 3.2 ppm (a major component of seminal plasma and sperm, see below) was also integrated and tracked through each stage of DGC and a smaller reduction in the choline concentration in ‘40%’ and ‘80%’ sperm (>55-fold and >100-fold, respectively) was also observed (Fig. 3b). Notably, the concentration of choline was greater in the spectrum from the two sperm populations, rather than the spectrum for the ‘wash’ (Fractions G and H) suggesting that it was associated with the sperm themselves.

To estimate the amount of seminal plasma found within the ‘40%’ and ‘80%’ sperm populations, the bin containing the fumarate peak in the seminal plasma spectrum was scaled to the fumarate peak in the sperm spectrum. This found 3.0 ± 0.9% of seminal plasma components in ‘80%’ sperm compared to 15.6 ± 4.5% in ‘40%’ (sum of bin integrals, *n* = 4). In addition, the MRS signal from 50% Percoll/PBS solution was also fitted to each sperm spectrum to estimate its remaining content and this showed that Percoll represented the larger component in both sperm spectra; 60 ± 5% of the ‘40%’ sperm (Fraction I, see example Supplementary Fig. S3) and 59 ± 11% of the ‘80%’ sperm (Fraction J, see example Supplementary Fig. S4).

#### Experiment 3: DGC with an additional centrifugation step

**Figure 4:**
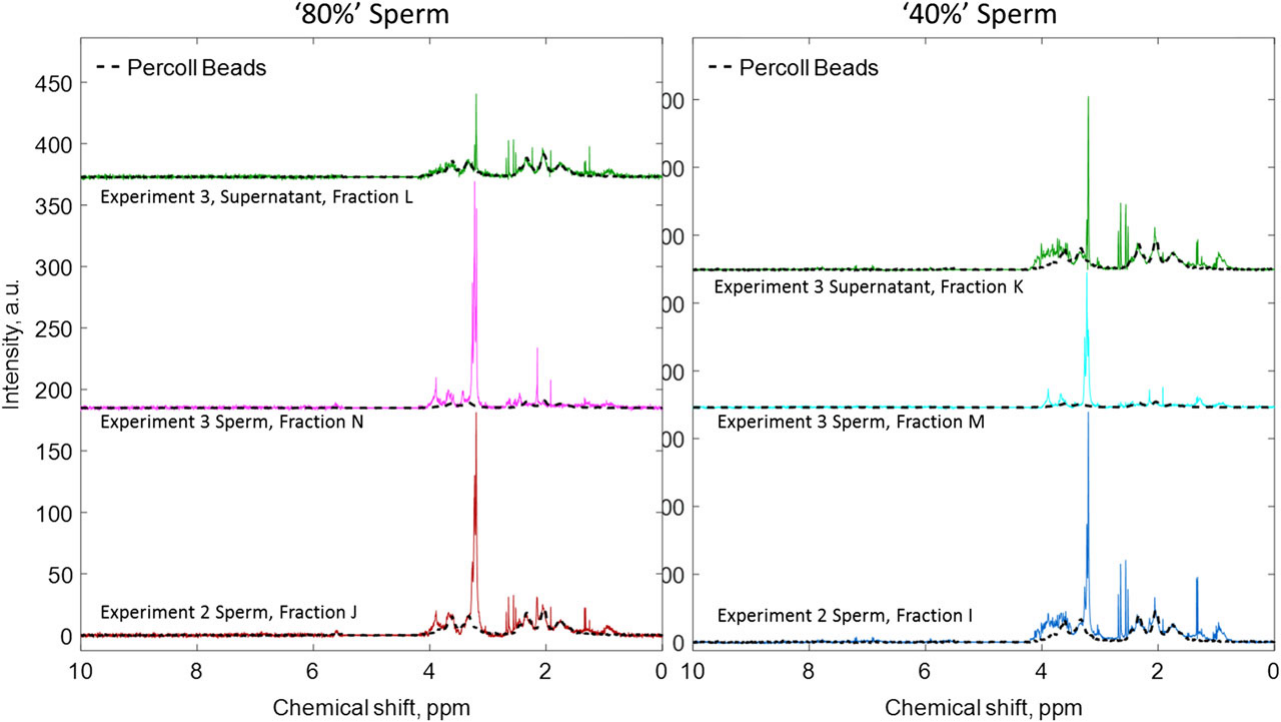
Representative ^1^H MRS spectra of the sperm fraction after the Experiment 2 washing protocol and Experiment 3 centrifuge protocol (80%, left; 40% right). The supernatant from the Experiment 3 centrifuge sperm fraction is also shown, along with a fitted Percoll beads spectrum (black dashed line) to indicate the quantity of the respective density gradient solution removed by the additional washing step.

To further remove Percoll (and any other remnants of seminal plasma from the sperm spectrum) an extra centrifugation step was added to the sperm washing protocol. The spectra obtained from ‘40%’ and ‘80%’ sperm (Fractions M and N), along with the supernatant obtained from the additional washing step (Fraction K for ‘40%’ sperm supernatant and Fraction L for ‘80%’ sperm supernatant) are shown in Fig. 4. Critically, following this method, the Percoll content of the ‘80%’ sperm population was reduced from 60 ± 3% in the first washing step to 26 ± 1% in the second (sum of bin integrals, *n* = 3); similarly for ‘40%’ sperm the Percoll content was reduced from 60 ± 2% to 31 ± 1% (sum of bin integrals, *n* = 3, see example Supplementary Fig. S5). Fitting a representative seminal plasma spectrum from Experiment 3 to ‘80%’ sperm (Fractions J and N) showed there was almost no change in seminal plasma content 8.5 ± 4.6% and 6.5 ± 1.6%, respectively (sum of bin integrals, *n* = 3). Conversely, for ‘40%’ sperm the additional washing step reduced seminal plasma content from 17.5 ± 7.2% (Fraction I) to 4.9 ± 1.1% after washing (Fraction M, sum of bin integrals, *n* = 3, see example Supplementary Fig. S6).

Experiment 3 recovered 30 ± 4% of the total sperm (from Fractions I, J, M and N), with no difference between the number of recovered sperm in the Experiment 2 and Experiment 3 centrifuge samples, both 15 ± 3%. Importantly the progressive motility was not significantly different between the two centrifugation steps (data not shown). With respect to its original concentration in semen the proportion of seminal plasma in ‘80%’ sperm was estimated to be 1/9100 (Fraction N, mean, range 1/4700–1/50 000, sum of bin integrals, *n* = 3) and 1/16 000 for ‘40%’ sperm (Fraction M, mean, range 1/7700–1/49 000, sum of bin integrals, *n* = 3).

### Sperm concentration required for MRS

**Figure 5:**
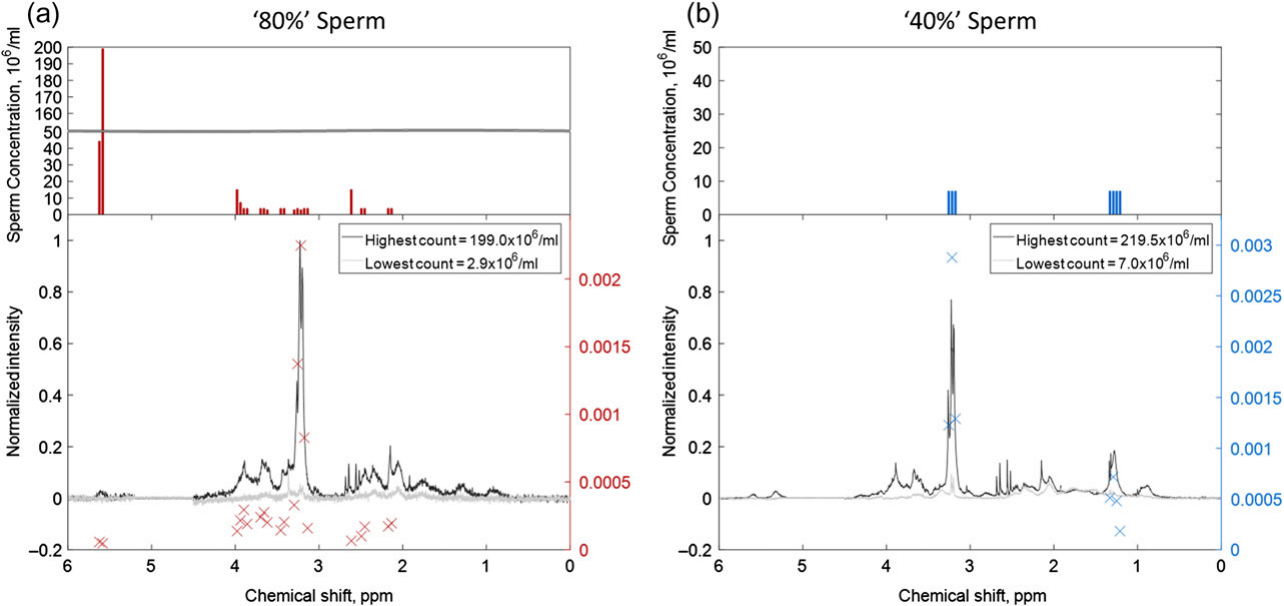
Correlation of spectral bins versus sperm count. The bottom portion of the figure shows 80% sperm (**a**), and 40% sperm (**b**), spectra from the lowest (light gray) and highest (dark gray) sperm count. Superimposed on the spectra are the locations of significant Pearson correlation coefficients between the bin integral and sperm count, red (80%) and blue (40%) ‘x’ marker. The right hand axis indicates gradient of the correlation associated with each marker. In the upper portion of the figure shows the lowest sperm concentration for each correlated bin with a signal to noise ratio (SNR) > 3:1.

To identify which metabolite peaks were associated with sperm, 31 matched ^1^H spectra were acquired from the ‘40%’ and ‘80%’ sperm populations (Fractions I and J), processed as outlined above, and bin integrals (0.04 ppm width) correlated against the sperm concentration in the MRS tube. For a range of sperm concentrations from 2.9 to 199 × 10^6^/ml for the ‘80%’ sperm population, 21 correlations were found with a coefficient of determination, *r*^2^, greater than 0.25 and significance, *P* < 0.01 (see Fig. 5a and Supplementary Material for fitting parameters and example plots of bin integral versus sperm concentration, Supplementary Fig. S7). Correlated bins included choline/glycerophosphocholine (GPC), 3.22–3.26 ppm, as well as citrate, 2.49–2.61 ppm and acetylcarnitine, 2.17 ppm. The sperm concentration range required to produce a peak with a SNR > 3:1 was from 2.9 × 10^6^/ml for choline/GPC (the most intense peaks observed in the spectrum) to 199 × 10^6^/ml for a peak assigned as unsaturated aliphatic carbon chain (5.58–5.62 ppm). For the ‘40%’ sperm population (with washed sperm concentrations from 7.0 to 220 × 10^6^/ml), only seven bin correlations were found (Fig. 5b). In ‘40%’ sperm populations there was also a notable correlation between sperm concentration and the choline/GPC peaks from 3.22 to 3.26 ppm. The lowest ‘40%’ sperm concentration was 7 × 10^6^/ml and this was sufficient to produce peaks for all the correlated bins with a SNR greater than 3:1. Using the spectrum acquired at 7 × 10^6^ sperm/ml the SNR was calculated for the most intense peaks within each correlated bin chemical shift range. These SNR values were then used to estimate the theoretical sperm concentration that would produce a peak SNR of 3:1 within each correlated bin range and this was found to be ~3 × 10^6^/ml.

### Comparison of ^1^H MRS spectra from ‘40%’ and ‘80%’ sperm

**Figure 6:**
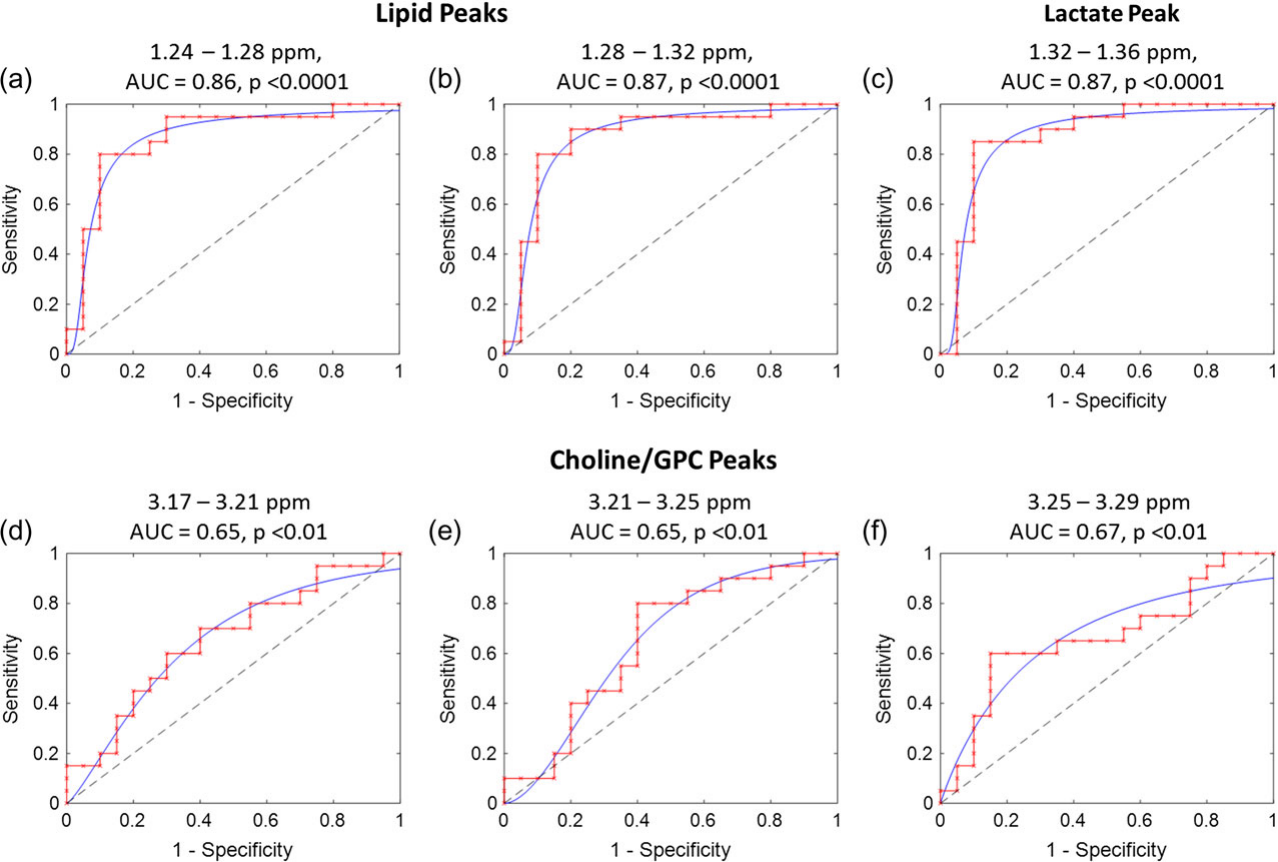
ROC curve analysis of ^1^H MRS spectrum identified as being significantly different between ‘40%’ and ‘80%’ sperm populations by two-way ANOVA. Six spectrum bins were found using two-way ANOVA (spectrum bins and sperm populations) in two distinct regions of the ^1^H MRS spectrum; choline/GPC (3.17–3.29 ppm), lactate (1.32–1.36 ppm) and lipid (1.24–1.32 ppm). The ability of each of these bins to discriminate between sperm populations was analyzed using ROC curves; Panels (**a**)–(**c**) lactate/lipid region, Panels (**d**)–(**e**) choline/GPC.

The ability of ^1^H MRS to discriminate between the ‘40%’ and ‘80%’ sperm recovered from DGC (Experiment 3) was tested by a two-way ANOVA. This showed that there were statistically significant differences between the spectral bins (*P* < 0.0001), ‘40%’ and ‘80%’ sperm populations (*P* < 0.0001) as well as a significant interaction term between spectral bins and ‘40%’ and ‘80%’ sperm populations (*P* < 0.0001). Applying a Bonferroni multivariate comparison to the data identified six bins where metabolite integrals were significantly higher in the ‘40%’ sperm compared to ‘80%’ sperm populations. These were for chemical shift regions associated with lactate (1 bin, 1.32–1.36 ppm, *P* = 0.003), lipid peaks (2 bins, 1.24–1.32 ppm, *P* < 10^−27^) and choline/GPC (3 bins, 3.17–3.29 ppm, *P* < 10^−11^), see Table I for mean ± SE values. The bin integrals from the identified significant locations where then used to discriminate between ‘40%’ and ‘80%’ sperm populations by ROC analysis. This showed that lactate and lipid peak regions were highly predictive of sperm population type (AUC 0.86–0.87), see Fig. 6. The ROC for choline/GPC peaks (3.17–3.29 ppm) were also significant but showed less predictive power (AUC 0.65–0.67). A Student's *t*-test found no significant differences between these populations in terms of sperm concentration or progressive motility.

**Table I:** Bin positions identified as being significantly different between ‘40%’ and ‘80%’ sperm by two-way ANOVA (*n* = 20). Multivariate analysis with Bonferroni correction was applied to determine *P* values for individual bins.

Bin, ppm	‘40%’ sperm	‘80%’ sperm	Multivariate comparison *P* value
	Mean ± SE	Mean ± SE	
1.26	25.41 ± 1.08	6.58 ± 1.08	2 × 10^−29^
1.30	25.38 ± 1.08	6.93 ± 1.08	4 × 10^−28^
1.34	12.30 ± 1.08	3.90 ± 1.08	3 × 10^−3^
3.19	63.74 ± 1.08	48.45 ± 1.08	3 × 10^−18^
3.23	93.18 ± 1.08	68.86 ± 1.08	8 × 10^−51^
3.27	38.51 ± 1.08	26.19 ± 1.08	8 × 10^−11^

## Discussion

The data presented in this paper are to the best of our knowledge the first to show that it is possible to obtain ^1^H MRS spectra from live human spermatozoa and that ^1^H MRS can be used to discriminated between sperm in the ‘40%’ and ‘80%’ populations recovered from ejaculates with normal sperm concentration and motility. However, to obtain suitable spectra of sperm for ^1^H MRS analysis, three technical challenges had to be overcome.

First it was necessary to be able to efficiently remove sperm from seminal plasma. Since sperm represent a relatively minor component of semen (estimated at 1–5% by volume, Mortimer (1994)) there is the potential for MRS signals from molecules in seminal plasma to dominate those obtained from sperm. To achieve this successfully, three commonly used sperm washing techniques were used and it was shown that DGC with an additional washing step (Experiment 3) allowed MRS spectra of sperm to be obtained with a SNR > 3:1 with minimal levels (<0.01%) of contamination from seminal fluid components. Although sperm washing is ordinarily a relatively straightforward procedure, this is a greater number of washing steps (and consequently centrifugation steps) than is commonly used to prepare sperm for assisted conception.

Second, for successful MRS of sperm, it was necessary to perform sperm washing using media and reagents which themselves did not contribute significantly to the ^1^H MRS signal. Commercial media and reagents commonly used in assisted conception for washing sperm often contain molecules (e.g. glucose, lactate) added by the manufacturer, presumably to provide energy substrates for sperm during the sperm preparation process. Initial experiments with commercially available products proved unsuccessful as the additional media derived components, which were visible on the ^1^H MRS spectra (data not shown), obscured the sperm derived peaks to an unacceptable degree. Therefore, PBS was chosen because it had been shown to be sufficient to support human sperm viability (Amaral *et al.*, 2011), in addition to exhibiting only a single water ^1^H MRS peak that is removed from the spectrum during data acquisition. However, for the gradient, we chose Percoll (Pertoft *et al.*, 1978) because it was relatively inexpensive and it could be successfully diluted with PBS to give the correct 40%/80% ratio for DGC. Despite this, we were still able to detect a signal from Percoll in the spectra after washing, but this was reduced to ≤26% for ‘80%’ sperm and ≤31% for the ‘40%’ sperm fractions after the second centrifugation step of Experiment 3 (Fig. 4). However, although Percoll was used successfully in assisted conception for many years, it is no longer licensed for human use (see Mortimer (2000) for a discussion of this issue). Therefore, if we were ever to develop MRS as a clinically relevant technique, where the sperm might be used therapeutically after scanning, then an alternative MRS silent compound would have to be sought or developed to prepare the density gradients. However, for the purposes of this study, the use of Percoll was sufficient to show that it is possible to wash sperm for MRS analysis.

Third, it was necessary to find a washing method which provided sufficient sperm for MRS scanning and which at the same time did not have a deleterious effect on sperm viability/function. At the start of these experiments, it was not at all clear whether sufficient live sperm could be recovered from a single ejaculate to generate an MRS signal of sufficient intensity. For example, in the experiments by Paiva *et al.* (2015), pooled samples of identical ejaculate phenotypes containing 150 million sperm were lyophilized for MRS analysis. If the same number of live sperm were required for the approach followed in this paper, then this would seriously undermine the clinical value of MRS as it would only be possible to perform with ejaculates with very high sperm counts. However, based on the work presented here we estimate the minimum sperm concentration required to produce a ^1^H MRS peak greater that 3:1 SNR (defined as the minimum acceptable level in this study) is ~3 × 10^6^/ml. Moreover, we conclude that the additional washing step required to prepare sperm did not result in a significant change in the sperm concentration and motility suggesting that this is a reasonable step to take. As such, this may allow the opportunity to use MRS to examine sperm from men with oligozoospermia as well as those with astheno- or teratozoospermia (and combinations of all three).

The MRS system used in these experiments was a 9.4 T magnet with a broadband observe MRS probe. The scanning time of each washed sperm sample was 1 h 17 min, therefore potentially making this technology useful in future andrology practice. We envisage that hardware improvements such as use of a higher magnetic field, improved MRS probe detection coil geometry and cryo-cooled coils and amplifiers would improve the sensitivity of detection further and therefore potentially allow detection of MRS signals from lower sperm concentrations. Furthermore, by employing specialized glass MRS tubes, the absolute number of sperm needed for scanning could theoretically be reduced still further and permit the analysis of sperm obtained from oligozoospermic men. Clinical MRI scanning of living patients is restricted in the specific absorption rate of radio frequency radiation (<4 W/kg), and, to a much lesser extent, magnetic field exposure. We do not envisage sperm to be significantly affected by strong magnetic fields or by the radio frequency waves transmitted by the MRS scanning sequence used (estimated as <1.2 W/kg). However, this will need to be specifically investigated in future studies.

From the MRS data obtained in this study, we have identified some of the major molecules present in spermatozoa. These include choline/GPC, lipids and acetylcarnitine in one or both sperm populations recovered from DGC. Without specialist techniques, such as multi-dimensional MRS or isotopic labeling strategies, MRS will typically identify molecules of less than 10 000 Daltons (Clore and Gronenborn, 1997). Therefore, we assume that the spectra shown in this article represent soluble molecules associated with cell signaling or metabolism. However, until we have performed further studies, we need to be cautious about their significance.

Statistical analyses of the ^1^H spectrum from ‘40%’ and ‘80%’ sperm populations highlighted significant differences in lactate, lipid, choline and GPC peaks that were greater in the ‘40%’ sperm population compared ‘80%’. Phospholipids are a major component present in the outer cellular structure of sperm and lipid, choline and GPC identified may be associated with this structure (Tavilani *et al.*, 2007). Since the ‘40%’ sperm population generally has more morphological defects (Aitken and West, 1990), the differences in spectra may reflect ultrastructural differences in sperm structure (e.g. cytoplasmic droplets). Indeed, these differences in sperm structure are likely to be the reason sperm are separated by the DGC method. Other studies have found significantly altered levels of fatty acids in pelleted sperm derived from men diagnosed as normo- or asthenozoospermic, however, the nature of the difference depends heavily on the specific fatty acid being compared (Aksoy *et al.*, 2006). The resolution used by the binning method, 0.04 ppm, does not differentiate the type of lipid and further work would be required to elucidate the identity of individual fatty acids. Increased lactate in ‘40%’ sperm may represent altered energy metabolism via glycolysis in these sperm compared to those from the ‘80%’ fraction and additional studies using ^13^C labeled tracer molecules could highlight the relative importance of differing metabolic pathways in these sperm populations. Overall, differences in these peaks may result from metabolic differences between the two sperm populations and this may give a useful insight into the pathology of sperm dysfunction.

Our study is not without criticism and has several limitations. First, only three or four ejaculates were used to assess the efficacy of each sperm washing protocol and only three methods of sperm washing were examined. However, while other sperm washing methods are available (e.g. swim-up) we chose to use DGC as this is commonly used in assisted conception so that our results had the most relevance to clinical practice. Second, we are aware that some of the MRS peaks present in seminal plasma, or the supernatants of the sperm washing steps, could be derived from dead or dying sperm with leaky plasma membranes. For example, analysis of the supernatant recovered from Experiment 3 sperm centrifugation step (Fractions K and L in Fig. 1) revealed several metabolites, including choline, citrate and lactate. While these may have been derived from seminal plasma carried through the fractions, there is also the possibility that they have originated from sperm during the extra centrifuge step by either by active transport or because of damage to the sperm during sperm washing. Further studies are needed to quantify the extent to which soluble factors are exported from sperm. Third, we cannot exclude the possibility that some of the MRS peaks may be from somatic cells (e.g. leukocytes and epithelial cells) and that these are differentially fractionated by the DGC along with sperm. There were generally more non-sperm cells in fraction ‘M’ compared to ‘N’ but our CASA analysis shows that this was typically ≤2% (i.e. ≤2 non-sperm cells per 100 sperm). The relationship between cell size, type, metabolism and MRS spectrum is not straightforward and further work is required to determine the contribution non-sperm cells make to the sperm MRS spectrum and whether we have to take more account of this in future experiments. Finally, the estimated minimum sperm concentration required for MRS is specific to the hardware used in our study and may be different in other spectrometers.

In conclusion, in this paper we show that ^1^H MRS can provide information about the molecules present in live human sperm and we propose that this offers the opportunity to study their relative importance and how they relate to sperm function. This may in turn lead to the development of new diagnostic tests or ultimately novel treatments for male factor infertility.

## Supplementary Material

Supplementary Data

## References

[gax025C1] Aitken RJ, West KM Analysis of the relationship between reactive oxygen species production and leucocyte infiltration in fractions of human semen separated on Percoll gradients. Int J Androl 1990;13:433–451. 1965724 10.1111/j.1365-2605.1990.tb01051.x

[gax025C2] Aksoy Y, Aksoy H, Altinkaynak K, Aydin HR, Ozkan A Sperm fatty acid composition in subfertile men. Prostaglandins Leukot Essent Fatty Acids 2006;75:75–79. 16893631 10.1016/j.plefa.2006.06.002

[gax025C3] Amaral A, Paiva C, Baptista M, Sousa AP, Ramalho-Santos J Exogenous glucose improves long-standing human sperm motility, viability, and mitochondrial function. Fertil Steril 2011;96:848–850. 21839434 10.1016/j.fertnstert.2011.07.1091

[gax025C4] Clore GM, Gronenborn AM NMR structures of proteins and protein complexes beyond 20,000 M(r). Nat Struct Biol 1997;4:849–853. 9377157

[gax025C5] De Jonge C Semen analysis: looking for an upgrade in class. Fertil Steril 2012;97:260–266. 22289285 10.1016/j.fertnstert.2011.12.045

[gax025C6] Dreanno C, Seguin F, Cosson J, Suquet M, Billard R ^1^H-NMR and (31)P-NMR analysis of energy metabolism of quiescent and motile turbot (Psetta maxima) spermatozoa. J Exp Zool 2000;286:513–522. 10684575 10.1002/(sici)1097-010x(20000401)286:5<513::aid-jez9>3.0.co;2-5

[gax025C7] Elder K, Dale B In Vitro Fertilization, 2nd edn Cambridge: Cambridge University Press, 2000.

[gax025C8] Emwas AHM, Salek RM, Griffin JL, Merzaban J NMR-based metabolomics in human disease diagnosis: applications, limitations, and recommendations. Metabolomics 2013;9:1048–1072.

[gax025C9] Gupta A, Mahdi AA, Ahmad MK, Shukla KK, Bansal N, Jaiswer SP, Shankhwar SN A proton NMR study of the effect of Mucuna pruriens on seminal plasma metabolites of infertile males. J Pharm Biomed Anal 2011a;55:1060–1066. 21459537 10.1016/j.jpba.2011.03.010

[gax025C10] Gupta A, Mahdi AA, Ahmad MK, Shukla KK, Jaiswer SP, Shankhwar SN ^1^H NMR spectroscopic studies on human seminal plasma: a probative discriminant function analysis classification model. J Pharm Biomed Anal 2011b;54:106–113. 20719458 10.1016/j.jpba.2010.07.021

[gax025C11] Hamamah S, Seguin F, Bujan L, Barthelemy C, Mieusset R, Lansac J Quantification by magnetic resonance spectroscopy of metabolites in seminal plasma able to differentiate different forms of azoospermia. Hum Reprod 1998;13:132–135. 9512244 10.1093/humrep/13.1.132

[gax025C12] Hung PH, Froenicke L, Lin CY, Lyons LA, Miller MG, Pinkerton KE, VandeVoort CA Effects of environmental tobacco smoke in vivo on rhesus monkey semen quality, sperm function, and sperm metabolism. Reprod Toxicol 2009;27:140–148. 19159676 10.1016/j.reprotox.2008.12.007

[gax025C13] Huser T, Orme CA, Hollars CW, Corzett MH, Balhorn R Raman spectroscopy of DNA packaging in individual human sperm cells distinguishes normal from abnormal cells. J Biophotonics 2009;2:322–332. 19373853 10.1002/jbio.200910012

[gax025C14] Jayaraman V, Ghosh S, Sengupta A, Srivastava S, Sonawat HM, Narayan PK Identification of biochemical differences between different forms of male infertility by nuclear magnetic resonance (NMR) spectroscopy. J Assist Reprod Gen 2014;31:1195–1204. 10.1007/s10815-014-0282-4 PMC4156941 24965760

[gax025C15] Lin CY, Hung PH, VandeVoort CA, Miller MG ^1^H NMR to investigate metabolism and energy supply in rhesus macaque sperm. Reprod Toxicol 2009;28:75–80. 19490998 10.1016/j.reprotox.2009.03.005

[gax025C16] Lynch MJ, Masters J, Pryor JP, Lindon JC, Spraul M, Foxall PJ, Nicholson JK Ultra high field NMR spectroscopic studies on human seminal fluid, seminal vesicle and prostatic secretions. J Pharm Biomed Anal 1994;12:5–19. 8161606 10.1016/0731-7085(94)80004-9

[gax025C17] Macleod J Human semen. Fertil Steril 1956;7:368–386. 13344556

[gax025C18] Marin S, Chiang K, Bassilian S, Lee WNP, Boros LG, Fernandez-Novell JM, Centelles JJ, Medrano A, Rodriguez-Gil JE, Cascante M Metabolic strategy of boar spermatozoa revealed by a metabolomic characterization. FEBS Lett 2003;554:342–346. 14623091 10.1016/s0014-5793(03)01185-2

[gax025C19] Mortimer D Practical Laboratory Andrology. New York, Oxford: Oxford University Press, 1994.

[gax025C20] Mortimer D Sperm preparation methods. J Androl 2000;21:357–366. 10819443

[gax025C21] Pacey AA Sperm, human fertility and society. In: Birkhead T, Hosken D, Pitnick S (eds). *Sperm Biology: An Evolutionary Perspective* Elsevier, 2009, 565–597.

[gax025C22] Paiva C, Amaral A, Rodriguez M, Canyellas N, Correig X, Ballesca JL, Ramalho-Santos J, Oliva R Identification of endogenous metabolites in human sperm cells using proton nuclear magnetic resonance ((1) H-NMR) spectroscopy and gas chromatography-mass spectrometry (GC-MS). Andrology 2015;3:496–505. 25854681 10.1111/andr.12027

[gax025C23] Patel AB, Srivastava S, Phadke RS, Govil G Arginine activates glycolysis of goat epididymal spermatozoa: an NMR study. Biophys J 1998;75:1522–1528. 9726954 10.1016/S0006-3495(98)74071-8 PMC1299827

[gax025C24] Patel AB, Srivastava S, Phadke RS, Govil G Identification of low-molecular-weight compounds in goat epididymis using multinuclear nuclear magnetic resonance. Anal Biochem 1999;266:205–215. 9888977 10.1006/abio.1998.2888

[gax025C25] Pertoft H, Laurent TC, Laas T, Kagedal L Density gradients prepared from colloidal silica particles coated by polyvinylpyrrolidone (Percoll). Anal Biochem 1978;88:271–282. 211867 10.1016/0003-2697(78)90419-0

[gax025C26] Sharma U, Chaudhury K, Jagannathan NR, Guha SK A proton NMR study of the effect of a new intravasal injectable male contraceptive RISUG on seminal plasma metabolites. Reproduction 2001;122:431–436. 11597307 10.1530/rep.0.1220431

[gax025C27] Tavilani H, Doosti M, Nourmohammadi I, Mahjub H, Vaisiraygani A, Salimi S, Hosseinipanah SM Lipid composition of spermatozoa in normozoospermic and asthenozoospermic males. Prostaglandins Leukot Essent Fatty Acids 2007;77:45–50. 17693070 10.1016/j.plefa.2007.07.001

[gax025C28] Tomlinson MJ Uncertainty of measurement and clinical value of semen analysis: has standardisation through professional guidelines helped or hindered progress. Andrology 2016;4:763–770. 27529487 10.1111/andr.12209

[gax025C29] WHO WHO Laboratory Manual for the Examination and Processing of Human Semen, 5th edn Geneva: World Health Organization, 2010.

