# Supplementary material for: 1H Magnetic Resonance Spectroscopy of live human sperm
Source: Mol Hum Reprod. 2017 May 23;23(7):441–51. doi: 10.1093/molehr/gax025 (PMC5909857; doi:10.1093/molehr/gax025)
Supplement: Supplementary Data [file supplementaryinformationv2.5.docx]

**Supplementary information**

**Matching component spectra to a target spectrum**

The proportion(s) of a particular sample constituent(s) contained within another target sample, e.g. residual seminal plasma within sperm, were estimated based on matching the respective spectrum profile for each constituent(s) to the target spectrum. In order to achieve this the following procedure was performed using custom Matlab software.

1. Normalization and alignment of spectrum

The scale of the target and component spectra were normalized by matching the intensity of a noise region of the spectrum containing no peaks between 10-11 ppm. Firstly, the mean value of this spectral region was subtracted from the total spectrum to correct for baseline offset. Secondly, the whole spectrum was divided by the interquartile range of the noise region. Subsequently, all of the component spectra were globally aligned to the target spectrum. The region of the spectrum containing a residual water peak, typically 4.2-5.2 ppm, was replaced with random numbers between -0.5 to +0.5.

2. Identification of peak regions

To avoid comparing regions of noise a masked template was created for each spectrum where spectral data points with an intensity less than three times the root mean squared (RMS) noise, determined from 10-11 ppm, were set to zero. The remaining non-zeroed spectral points were designated as peak regions. Only templated spectra were used in comparisons

3. Matching of component spectrum/spectra to target spectrum

A matrix of, up to 2, templated component spectra (CS) were scaled to the templated target spectrum (TS) to yield individual scaling factors, k, for each component spectrum.

[TS] = k_1-n_*[CS_1_,…, CS_n_]

The scaled component spectra were then summed to create a matched spectrum (MS).

$$\left[ \text{MS} \right]\text{= }\sum_{\text{i=1}}^{\text{n}\text{=2}} \text{k}_{\text{i}}\text{.}{\text{[}\text{CS}\text{]}}_{\text{i}}$$

4. Quantification of component spectrum in target spectrum

To determine how well the matched spectrum, and therefore the individual components, represented the target spectrum a spectrum binning method was used. TS, MS and k_i_.CS_i_ spectra were binned into 0.04 ppm regions from 0-11 ppm with the starting bin centered about the lactate peak at 1.33 ppm. The data points within each bin then were integrated and the ratio of MS to TS bin integrals, R(MS/TS), were expressed as a percentage. The percentage ratio of the scaled component spectra, k_i_.CS_i_, bin integrals to TS bin integrals, R(k_i_.CS_i_ /TS), were also determined for the relative contribution to each bin from the respective components, shown in Supplementary Figures as Panel (b). To determine the overall contribution of a sample fraction, e.g. Fraction ‘B’, to the target spectrum, e.g. Fraction ‘A,’ the bins were summed for the component spectrum, i.e. Fraction ‘B’, and target spectrum, i.e. Fraction ‘A’, and the sums expressed as a ratio. Regions with a poor fit were excluded from the summation by removing extreme outlier bins (defined as bin locations where R(MS/TS) had a value greater than three times the interquartile range of all the R(MS/TS) bin ratios). The median percentage bin value and interquartile range was also calculated as a measure of the quality of the overall fit of for the component spectrum, shown in Supplementary Figures as Panel (c).

**Correlation of ^1^H spectrum bin integrals to sperm concentration**

All spectra were normalized to the noise region between 10-11 ppm. Residual water signal in the spectra was removed using a spline fitting method to subtract the water peak from the spectrum. The spline fitting also corrected the baseline. The region of the spectrum containing a residual water peak, typically 4.2-5.2 ppm, was replaced with random numbers between -0.5 to +0.5. Spectra were binned at 0.04 ppm centered about the lactate peak at 1.33 ppm. A linear regression model was fitted for each bin versus sperm concentration in the MRS tube. Correlations used a minimum of 3 bin integrals that were greater than three times the RMS noise, as determined above, and only deemed valid if the correlation coefficient of determination, r^2^, was greater than 0.25, with a significance, p, less than 0.01. For each bin which met this inclusion criteria the signal to noise ratio (SNR) contained within the chemical shift region of the bin was calculated for all sperm concentrations. The spectrum with the lowest sperm concentration with a bin SNR > 3:1 was estimated as the limit of observability on the spectrometer used for our experiments. Tables S1 and S2 show the correlated bins for ‘80%’ and ‘40%’ sperm spectra respectively.

Table T1: 80% sperm spectra bin integral correlated with sperm concentration from Fraction J. Bin locations that produced a significant correlation (r^2^>0.25, p<0.01). Slope and intercept with the number of fitted data points (from a maximum of 31). Lowest Sperm concentration is the estimated lowest sperm concentration. * Lowest acquired sperm concentration, see main text for discussion.

| Chemical Shift, ppm | Lowest Sperm concentration, 10^6^/ml | Slope | Intercept | Fitted points | r^2^ | p |
| --- | --- | --- | --- | --- | --- | --- |
| 5.62 | 44.2 | 0.0001 | -0.0033 | 11 | 0.38 | 1.98E-04 |
| 5.58 | 199 | 0.0001 | -0.0013 | 13 | 0.26 | 3.27E-03 |
| 3.98 | 15.09 | 0.0003 | 0.0130 | 27 | 0.27 | 2.83E-03 |
| 3.94 | 7.3 | 0.0004 | 0.0192 | 28 | 0.46 | 2.57E-05 |
| 3.90 | 3.7 | 0.0006 | 0.0273 | 30 | 0.47 | 1.85E-05 |
| 3.86 | 3.7 | 0.0004 | 0.0248 | 30 | 0.35 | 5.01E-04 |
| 3.70 | 3.7 | 0.0005 | 0.0420 | 31 | 0.41 | 9.72E-05 |
| 3.66 | 3.7 | 0.0006 | 0.0517 | 31 | 0.47 | 2.30E-05 |
| 3.62 | 2.92* | 0.0004 | 0.0539 | 31 | 0.35 | 4.49E-04 |
| 3.46 | 3.7 | 0.0003 | 0.0189 | 30 | 0.53 | 3.52E-06 |
| 3.42 | 3.7 | 0.0004 | 0.0332 | 31 | 0.59 | 4.56E-07 |
| 3.30 | 2.92 | 0.0007 | 0.0632 | 31 | 0.47 | 2.22E-05 |
| 3.26 | 3.7 | 0.0027 | 0.0801 | 31 | 0.70 | 3.62E-09 |
| 3.22 | 2.92* | 0.0045 | 0.1607 | 31 | 0.81 | 7.87E-12 |
| 3.18 | 3.7 | 0.0017 | 0.0820 | 31 | 0.57 | 9.02E-07 |
| 3.14 | 3.7 | 0.0003 | 0.0291 | 31 | 0.40 | 1.44E-04 |
| 2.61 | 15.09 | 0.0001 | 0.0083 | 30 | 0.29 | 1.76E-03 |
| 2.49 | 3.7 | 0.0002 | 0.0319 | 31 | 0.30 | 1.43E-03 |
| 2.45 | 3.7 | 0.0003 | 0.0443 | 31 | 0.44 | 5.11E-05 |
| 2.17 | 3.7 | 0.0003 | 0.0314 | 31 | 0.43 | 6.01E-05 |
| 2.13 | 3.7 | 0.0004 | 0.0493 | 31 | 0.31 | 1.07E-03 |

Table T2: 40% sperm spectra bin integral correlated with sperm concentration from Fraction I. Bin locations that produced a significant correlation (r^2^>0.25, p<0.01). Slope and intercept with the number of fitted data points (from a maximum of 30). Lowest Sperm concentration is the estimated lowest sperm concentration. * Lowest acquired sperm concentration, see main text for discussion.

| Chemical Shift, ppm | Lowest Sperm concentration, 10^6^/ml | Slope | Intercept | Fitted points | r^2^ | p |
| --- | --- | --- | --- | --- | --- | --- |
| 3.26 | 7* | 0.0012 | 0.0569 | 30 | 0.57 | 1.47E-06 |
| 3.22 | 7* | 0.0029 | 0.1809 | 30 | 0.50 | 1.12E-05 |
| 3.18 | 7* | 0.0013 | 0.0700 | 30 | 0.52 | 6.57E-06 |
| 1.33 | 7* | 0.0005 | 0.0437 | 30 | 0.36 | 4.34E-04 |
| 1.29 | 7* | 0.0007 | 0.0189 | 30 | 0.45 | 4.66E-05 |
| 1.25 | 7* | 0.0005 | 0.0153 | 30 | 0.50 | 1.16E-05 |
| 1.21 | 7* | 0.0002 | 0.0181 | 30 | 0.32 | 1.08E-03 |
